# The Influence of Eggshell on Bone Regeneration in Preclinical In Vivo Studies

**DOI:** 10.3390/biology9120476

**Published:** 2020-12-18

**Authors:** Horia Opris, Cristian Dinu, Mihaela Baciut, Grigore Baciut, Ileana Mitre, Bogdan Crisan, Gabriel Armencea, Daiana Antoaneta Prodan, Simion Bran

**Affiliations:** Department of Maxillofacial Surgery and Implantology, University of Medicine and Pharmacy “Iuliu Hatieganu”, 400012 Cluj-Napoca, Romania; horia.opris@gmail.com (H.O.); gbaciut@umfcluj.ro (G.B.); ilmitre@yahoo.com (I.M.); bbcrisan@yahoo.com (B.C.); garmencea@gmail.com (G.A.); daiana.a.opris@gmail.com (D.A.P.); dr_brans@yahoo.com (S.B.)

**Keywords:** eggshell, guided bone regeneration, animal study, bone defect

## Abstract

**Simple Summary:**

The aim of this study is to review the available information on the use of avian eggshell as bone regeneration material. Five databases were searched up to October 2020. Animal studies with a bone defect model using eggshell as a grafting material were included. Risk of bias and the quality of the papers were assessed. Overall, a total of 581 studies were included in the study, 187 after duplicate removal. Using the inclusion and exclusion criteria 167 records were further excluded. The full text of the remaining 20 articles was assessed for eligibility and included in the review. There were different methods of obtaining eggshell for grafting purposes. Eggshell is a biocompatible grafting material, with bone formation capabilities. It forms new bone similar to other products currently in use in clinical practice. It can be combined with other materials to enhance its proprieties. Eggshell is a promising biomaterial to be used in bone grafting procedures, though further research is needed.

**Abstract:**

The aim of this study is to systemically review the available evidence on the in vivo behavior of eggshell as a guided bone regeneration substitute material. Five databases (PubMed, Cochrane, Web of Science, Scopus, EMBASE) were searched up to October 2020. In vivo animal studies with a bone defect model using eggshell as a grafting material were included. Risk of bias was assessed using SYRCLE tool and the quality assessment using the ARRIVE guidelines. Overall, a total of 581 studies were included in the study, 187 after duplicate removal. Using the inclusion and exclusion criteria 167 records were further excluded. The full text of the remaining 20 articles was assessed for eligibility and included in the qualitative and quantitative assessment synthesis. There were different methods of obtaining eggshell grafting materials. Eggshell is a biocompatible grafting material, with osteoconduction proprieties. It forms new bone similar to Bio-Oss and demineralized freeze-dried bone matrix. It can be combined with other materials to enhance its proprieties. Due to the high variability of the procedures, animals, production and assessment methods, no meta-analysis could be performed. Eggshell might be considered a promising biomaterial to be used in bone grafting procedures, though further research is needed.

## 1. Introduction

Guided bone regeneration (GBR) is the method used in oral surgery to increase the volume of available host bone in sites chosen for dental implant therapy [1]. The original concept that led to the biological principles of guided tissue regeneration were developed as a desire to regenerate lost periodontal tissues [2,3]. This principle has become golden standard in situations with an inadequate volume of bone where dental implants are planned [4].

Avian eggshell has been introduced for a while now in the maxillofacial reconstructive surgery due to its mineral composition similar to coral (95% CaCO_3_) [5]. Reports show issues with the healing, to be more exact fibrous union [5,6,7]. Some authors have tried to surface-modify the eggshell to enhance its proprieties [8]. It is expected that the use of a derived material from eggshell (a bioresorbable CaCO_3_) may have several advantages due to its availability and biodegradability [7,8].

Hence, the purpose of this systematic review was to assess the in vivo performance of a novel biomaterial derived out of avian eggshell in animal bone defects.

## 2. Materials and Methods

The protocol of the study was registered in the International prospective register of systematic reviews (PROSPERO ID CRD42020187327).

### 2.1. Protocol Development and Reporting Format

The review protocol was developed under the PRISMA guidelines [9]. The focused PICO (Population, Intervention, Comparison, Outcome) question was the following: in bone defects in experimental animal models, does the use of eggshell derived biomaterials improve new bone formation, compared to leaving the defect empty or filled with other commercially available products?

### 2.2. Eligibility Criteria

A priori, inclusion and exclusion criteria were defined. Only articles written in English were considered eligible. Regarding the study design, randomized control trials (RCTs), non-randomized control trials (non-RCTs) studies with split-mouth and parallel arms designs, were considered.

The inclusion criteria were the use of all animal studies with the bone defect model that uses guided bone regeneration with eggshell derived biomaterials, all sexes, species: Wistar rat, New Zealand rabbit, Sprague-Dawley rat, transgenic mice. The only type of included intervention was of the studies with guided bone regeneration procedures with eggshell derived biomaterials.

The exclusion criteria eliminated in vitro studies, human studies, in silico studies, reviews, meta-analyses, conference proceedings, book chapters, letters to the editor, technical notes, unclear or insufficient information for data quantification. Studies reporting ectopic models were also excluded.

### 2.3. Information Sources and Screening

An electronic search was conducted through five databases (PubMed, Cochrane, Web of Science, Scopus, EMBASE), to identify all in vivo studies published in English up to October 2020, using the following keywords: “eggshell” with the following terms “bone regeneration”, “guided bone regeneration”, “osseointegration”, “tissue regeneration”, “bone graft”, “bone healing”, “bone biology”, “bone substitute”, “bone repair”, “bone health”, “bone metabolism”.

A two staged screening was carried out. The screening of the titles and of the abstracts was performed in duplicate and independently by two reviewers. Full texts of eligible papers were obtained and reviewed independently by the same two reviewers. Secondly, articles meeting the inclusion criteria were assessed in full. Reasons for exclusion were also entered. Any disagreement between the reviewers was resolved through discussion, and in case a conclusion was not established, the third reviewer was consulted.

### 2.4. Data Collection

Characteristics of the included studies were extracted by two reviewers. The following data was taken out: author, year, country study design, study period, main objectives, animal type used, sample size, type of material used, intervention site, clinical and radiological assessment, biopsy (histology), follow-up, complications, excluded subjects, outcome.

### 2.5. Outcome Measures

Primary outcomes: New bone formation can be measured with different techniques such as histomorphometric analysis, radiographic analysis like computer tomography, micro-CT, standard radiograph and residual biomaterial.

Secondary Outcomes: any complications and adverse events related to the biomaterial used. The eggshell derived biomaterials and characterization were also investigated.

### 2.6. Quality Assessment and Risk of Bias Assessment

The quality of the studies was assessed independently by two reviewers (H.O. and G.A.), based on the ARRIVE (Animal in Research: Reporting In vivo Experiments) guidelines [10]. The items considered were the following: ethical statement, experimental procedures, experimental animals, randomization, allocation concealment, sample size calculation, completeness of information, blinding of the evaluator and financial conflict of interest.

SYRCLE’s risk of bias tool for animal studies [11] was used for the quantification of risk of bias in ten domains. All items could be judged as yes/no/unclear. Studies were considered at high risk of bias if at least two items were judged as “no”. Studies were judged as low risk of bias if at least seven items were judged as “yes”, and no item was judged as “no”. In other cases, the studies were considered at medium risk of bias. Two reviewers independently assessed the risk of bias for the included articles and if any disagreement occurred, a third reviewer intervened.

## 3. Results

### 3.1. Study Selection

**Figure 1:**
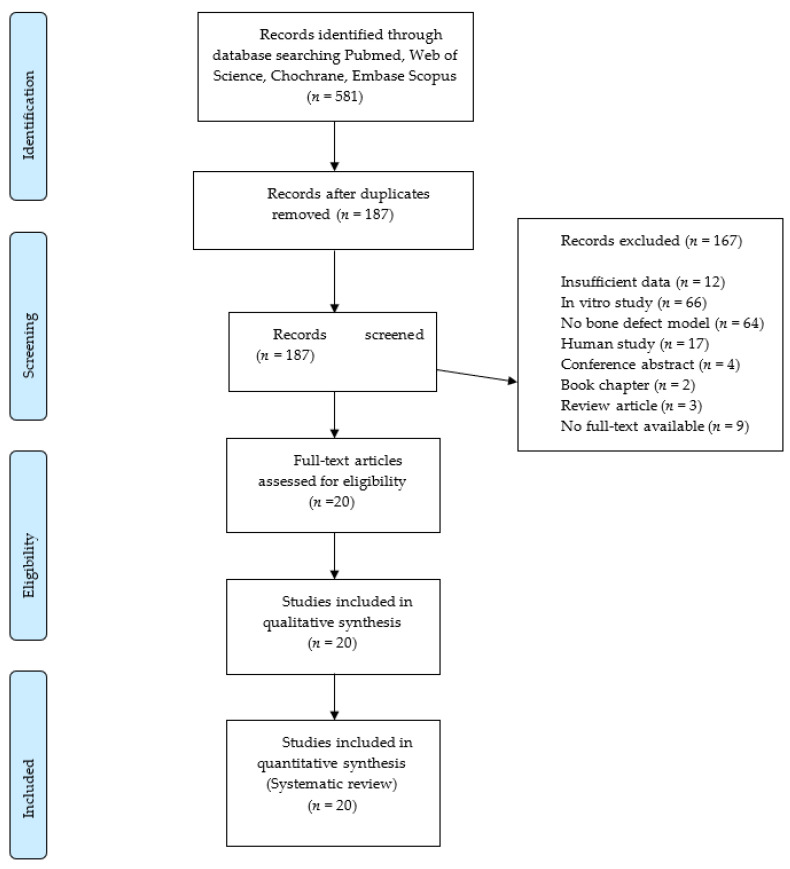
Flowchart of the article selection procedure.

The electronic search provided 518 articles that were reduced to 187 after the duplicate removal. No further articles were identified by manual search. Screening of titles and abstracts led to the exclusion of 167 records. The full texts of the remaining 20 articles were obtained. These papers were analyzed systematically and quality-wise. The flow diagram of the search results is shown in Figure 1.

### 3.2. Study Characteristics

Only qualitative data was extracted from each study and it was synthetized in analytic tables. Table 1 and Table 2 summarize the definition of the critical sized defect in the selected studies. In eight of the included papers Wistar Rats were used [5,6,12,13,14,15,16,17], six studies used Sprague-Dawley rats [8,18,19,20,21,22] and seven New Zealand rabbits [6,23,24,25,26,27,28]. The calvaria critical-sized bone defect was used in 90% of the included papers to assess new bone formation (Table 1 and Table 2).

None of the studies included the use of eggshell scaffold with bone marrow-derived mesenchymal stem cells. Non-Resorbable membranes such as ePTFE (expanded polytetrafluoroethylene) were used for GBR in two studies [13,14]. Collagen resorbable membranes were used for guided bone regeneration in one study [17]. A paper [23] attempted to use eggshell membranes as a resorbable alternative in bone grafting. Histology was used in all studies to assess bone healing (*n* = 20), histomorphometry in 30% of papers (*n* = 6), microCT in 25% of papers (*n* = 5) and contact radiograph in 30% of papers (*n* = 6). Other less often used investigations include immunohistochemistry (*n* = 1) and fluorescent labeling (*n* = 1). Follow-ups ranged from 2 to 24 weeks. A single observation interval was reported for eight of the included studies [5,6,12,13,15,22,23,28], while the rest had multiple observation points.

Because chemical composition and processing technology are considered important factors for determining the benefit of using the biomaterial, they were analyzed and summarized in Table 3 for the hen and ostrich eggshell. The most employed methods of assessing the materials are scanning electron microscopy (55%), x-ray diffractometry (40%), Fourier-transform infrared spectroscopy (30%) and energy-dispersive x-ray spectroscopy (10%).

A variety of production methods are described for the eggshell grafts, starting with eggshell blocks [6,15]. 75% of the included studies produced the biomaterial by crushing and milling eggshell into a powder. Calcination is being used in 35% of the cases to treat eggshell to improve its bone regeneration capabilities (*n* = 7). Hydrothermally treating the grafts greatly improves the porosity and structure of the eggshell [8,18]. A study [25] designed the use of silk fibroin scaffolds with eggshell to try to increase the proprieties of the biomaterial. Others [21] used a combination of carrageenan gel, xanthan gum gel to manufacture new biomaterials. Pure brushite cement was used [17] to find the best cement as bone augmentation material.

One study employed a commercially available nutritional supplement derived from eggshells used to promote healthy bones [28]. One study used a scaffold with carboxymethyl chitosan and BMP2 to enhance bone regeneration [12]. The ostrich eggshells were used in particles ranging from 20 to 1500 µm size. Sterilization of the products was done by either autoclaving, gamma irradiation or ethylene oxide.

### 3.3. Studies in Rabbits—Main Features

The characteristics and the main features of the studies in rabbits are summarized in Table 2. The included papers reported uneventful healing outcomes and no relevant adverse reactions. All included studies used the calvaria bone defect model with a range of 6 to 15 mm diameter, all except one [24] being full thickness. The number of defects per animal varied from one to six. Ostrich eggshell particles of different sizes were used in two studies [23,24]. No inflammatory reaction was noted in either study, bone regeneration seems to begin from the margins of the defect. Smaller graft particles resorb faster than larger ones. When compared to the demineralized bone matrix (DBM) graft, ostrich eggshell resorbs slower and produces less bone than DBM. Ostrich eggshell block was used in one study as an interposition graft [6]. When analyzed after the follow-up period, the interposition grafts seem to be delineated from the surrounding bone with no signs of remodeling.

Another study [26] compared the eggshell hydroxyapatite with the synthetic hydroxyapatite in the rabbit model and found no difference between the groups regarding new bone formation, both having low inflammatory response.

One of the papers [28] employed a nutritional supplement commercially available product from eggshell as a guided bone regeneration material—Membrell’s Bonehealth Plus. After two weeks of follow-up there was no sign of inflammatory response, the graft tissue was completely resorbed and there was deposition of newly formed bone. Eggshell particles combined with silk fibroin in a rabbit calvaria bone defect model showed similar results to eggshell particles alone regarding new bone formation [25].

### 3.4. Studies in Rats—Main Features

The characteristics and the main findings of the studies in rats are provided in Table 1. None of the studies reported adverse effects of the biomaterials used. To evaluate the bone regeneration capabilities of the biomaterial, 11 of the studies used the calvaria model, two studies [5,21] worked on a mandible/maxilla defect model and one used a periodontal bone defect [16]. Defect size ranged from 4 to 5 mm diameter.

Only one study [5] employed the use of the split mouth model, with one defect left empty and one with the graft. The biomaterial was found to have a uniform distribution in the defect site and be surrounded by a thin fibrous layer.

Dupoirieux et al. [6] used an ostrich eggshell implant as an interposition graft to compare it to an empty defect. The in vivo model showed that the block had little resorption and there was a radiolucency surrounding the implant, mainly formed of fibrous tissue.

Two studies by Dupoirieux et al. [13,14] used expanded polytetrafluoroethylene (ePTFE) membranes for guided bone regenerating in the calvaria bone defect model. The first paper [13] used carboxymethyl cellulose and pentosan polysulphate alongside eggshell powder. Bone healing started from the margins with centripetal bone formation. The group containing eggshell showed the most resorption with interposition fibrous tissue. In the second study [14] the non-resorbable membranes were used and compared to a periosteal graft and to eggshell powder. The results show no resorption of the eggshell powder and no complete closure with bone in this group.

Only one study [16] used a split mouth periodontal defect model to compare the eggshell particles to demineralized freeze-dried bone matrix (DMBM), with the use of collagen membranes on all grafts. The tissues healed with new bone forming from the margins of the defect with more connective tissue in the graft group.

Another paper [12] used eggshell particles to create a scaffold for bone morphogenetic proteins 2 (BMP2). The results were excellent with new bone formation, complete cover of the defect and enhanced bone formation capabilities in the scaffold group.

Using ostrich eggshell powder and an eggshell implant to compare them to DMBM, Uygur et al. concluded that there was no difference regarding new bone formation, but the DMBM still produces significantly more new bone [15].

Three studies [8,18,19] compared the eggshell with Bio-Oss. Park et al. [8] compared surface-modified eggshell particles with calcium phosphate with Bio-Oss. In the Bio-Oss group, bone healing was incomplete and there was no resorption of the graft. In the eggshell group complete bony closure was seen (40%), whereas in the surface-modified eggshell lot complete bone bridging was observed more often (80%). The authors have reported superior new bone regeneration in the surface-modified eggshell particles group. In another publication [19] the eggshell particles were compared with the Bio-Oss and eggshell hydroxyapatite enhanced with calcium sulphate. The most newly formed bone was with the nanohydroxyapatite derived from eggshell. Comparison of the use of deproteinized eggshell particles with hydrothermally treated eggshell particles and Bio-Oss revealed that there is new bone formation for all the groups, but the hydrothermally treated eggshell powder had almost complete bone healing with significantly greater new bone formation, even than Bio-Oss [18].

Another paper combined the use of eggshell derived biomaterials with brushite cement to produce an eggshell brushite cement [17]. This guided bone regeneration model uses collagen membranes on all grafted sites. The newly developed material resorbs faster than pure brushite and the newly formed bone starts from the margins of the defect and is present in a greater quantity. The pure brushite on its own is surrounded by inflammatory cells, unlike the eggshell brushite cement.

In another paper, Biocoral is compared to microroughened ostrich eggshell particles and calcium phosphate coated ostrich eggshell particles [22]. There were no significant differences between the grafted groups with new bone being formed from the margins of the defect and around the particles in the center. It seems that the Biocoral and the eggshell derived biomaterial have both similar bone regeneration proprieties.

Only one study [20] compared the eggshells to synthetic hydroxyapatite in which more new bone resulted especially around the grafted particles. Interestingly, around the synthetic hydroxyapatite there were more foreign body multinucleate cells. Comparing the bone volume on a microCT scan, the eggshell group showed significantly more volume than the synthetic hydroxyapatite.

Alternative biomaterials derived from eggshell were produced and a combination of carrageenan gel and xanthan gel have been used in the bone defect model [21] to compare it with the eggshell particles. The combination between the eggshell particles and carrageenan gel determined complete defect healing at the end of the study period.

### 3.5. Quality and Risk of Bias Assessment

A quality assessment was undergone according to ARRIVE guidelines [10] with nine scoring criteria (Supplementary Table S1). The result of the evaluation is provided in the Supplementary Table S2. A percentage of 70% (*n* = 14) of all of the studies reported data on the ethical statements with the exception of five which have failed to provide any information [5,6,13,14,18].

In all studies complete and adequate information was reported regarding the experimental procedures and the experimental animals. Regarding the allocation concealment and blinding the evaluator, the information was incomplete in all the papers. Most studies (70%) have incomplete financial information and lack the financial acknowledgment except for 6 studies (30%). Eight of the research papers (40%) randomly assigned the animals in different treatment groups. Only one [16] (5%) calculated the sample size using G Power software.

The risk of bias assessment of the selected studies using SYRCLE tool [11] is provided in the Supplementary Table S3. None of the studies have reported on blinding of the care giver, the investigator, or the assessor. Using the tool mentioned we assessed all papers to have a high risk of bias.

## 4. Discussion

The aim of this systematic review was to investigate the role of eggshell biomaterials used in guided bone regeneration in critical sized bone defects on experimental animal models compared to an empty defect or other filling materials. Overall, the results show that eggshell induces new bone formation compared to an untreated empty defect. There was little spontaneous bone regeneration in the control groups. When DMBM [16] or Bio-Oss [8,18,19] was used, similar results concerning new bone formation was observed.

Studies conducted on animal models demonstrated the beneficial use of bioceramic scaffolds in guided bone regeneration procedures. The architecture of the material is the key to conduct proper bone healing. An interconnected porous structure similar to natural bone should be present facilitating cell ingrowth, proliferation, and differentiation [29]. The biomaterial should possess adequate mechanical proprieties necessary for a functional loaded area such as the alveolar bone [30].

Resorption rate of the biomaterial should be matched with the osteogenesis rate occurring in the new bone [31]. One way of modifying these rates is to use composite materials alongside biodegradable polymers [32].

Recent studies using PLA (Polylactic acid) and human bone marrow stem cells as a bone substitute on a rodent calvaria bone defect model showed good results with new bone formation at 8 and 16 weeks with no residual material. The groups containing stem cells showed more bone formation at the end of the observation time [33]. Another study using the same in vivo model, compared PLA+ HA (hydroxyapatite) with demineralized bone matrix (DBM) and beta tricalcium phosphate. The beta tricalcium phosphate groups showed more new bone formation followed by the PLA+ HA and DBM groups [34].

Comparison of the included articles with human studies using eggshell as a bone regeneration substitute material shows similar results concerning bone regeneration, resorption rate and lack of immune response of the host [7]. The human studies use different bone defect model (apicectomy, cystectomy, third molar extraction site), but none use clearly defined guided bone regeneration model which does not heal spontaneously [35].

Other methods of producing biomaterials derived from eggshell can be the use of femtosecond laser processing to produce calcium carbonate 3D nanofibrous structures from eggshells [36]. Arslan et al. [37] used an in vitro model to produce a bio composite scaffold from eggshell waste, a collagen—keratin—nanohydroxyapatite with osteoinductive proprieties.

Polyether ether ketone (PEK)—biphasic bioceramic was used in a rabbit bone regeneration model with vascular endothelial growth factor with very good results at the end of the observation period with a significant new bone formation [38].

A combination of nanohydroxyapatite derived from eggshell enhanced with ZrO_2_ and Al_2_O_3_ was researched by Naga et al. [39] to increase porosity and decrease bulk density thus increasing the bone regeneration proprieties of the material. Further research is needed for this material using a bone defect model to test the in vivo proprieties.

The studies included with the rabbit model all have the calvaria bone defect. Other research has used other sites such as the mandibular bone [38,40], femoral defect [32] and radial defect [41].

The most frequent reason for exclusion in the systematic review was the lack of a bone defect model (64 studies excluded) and the fact that the papers did not present experimental models, but in vitro studies (66 papers excluded). Numerous eggshell biomaterials have been extensively developed and tested in vitro, but few studies developed in vivo bone defect models to test the tissue reaction and the bone formation.

In the included studies no extensive physico-chemical and mechanical characterization was undertaken. Regarding the fabrication of the biomaterials, summarized in Table 3, milling, calcination and hydrothermal treatment were the main procedures implied in fabrication. One study [28] used a commercially available nutritional supplement derived from eggshell as a bone regeneration material which was not indented for such specific use.

There were no studies on other animals closer to the human, like dogs, pigs, sheep, non-human primates, which could provide better insight due to the resemblance to the human metabolism. None of these studies included in the search had a bone defect model to match the inclusion criteria for this review.

Regarding the incorporating active molecules, only one study [12] achieved incorporating BMP2 into the eggshell graft with good results of newly formed bone.

The xenogenic bone substitutes of hydroxyapatite have been manufactured extensively and can be of synthetic origin, derived from corals or algae, or originated from natural bone mineral. It is considered that this material offers biocompatibility and osteoconduction properties (scaffold for the new bone formation). Depending on the particle size and the three-dimensional structures, it can exhibit different integration and resorption rates [42]. When used in alveolar ridge preservation, they offer good width and interproximal bone preservation [43]. Although the production and development are very different from one study to another, eggshells generally showed good biocompatibility, slow resorption rate (in inverse relationship with the size of the particles), osteoconductive proprieties (bone generally tends to heal from the margins of the defect as the graft resorbs).

The eggshell has several other uses in the medical field and the most important are the following: osteoporosis and joint mobility as a supplement, calcium supplement, orthopedics, cancer patients to boost muscle gain and hair thickening, sports nutrition to enhance performance [44].

Further limitations of this study are the fact that it includes only papers in English. Studies with ectopic tissue models were excluded. These could have provided only insight into the host soft tissue reaction to the grafts. When assessing the risk of bias all studies were evaluated as having high risk of bias according to the SYRCLE’s tool. There is a lot of debate about the lack of quality information in the animal experiments and because of which a representative risk of bias can be assessed. The risk of bias thus assessed with SYRCLE’s tool has to be viewed with its inherent limitations [45].

The calvaria model, which is the most often used in the papers included (85% of articles), is a very widespread model for the evaluation of bone regeneration materials. Nonetheless, its assessment does not offer insight to the biological response to the physiological biomechanical loading occurring in the alveolar bone [46].

There is an ongoing debate on what a critical size bone defect is for each model. In the calvaria model for rodents there are numerous advantages of a 5 mm diameter defect [47], with little spontaneous healing from the bone margins at 12 months [48]. Regarding the rabbit calvaria model, numerous studies have found that there is a direct correlation between the size of the defect and the time it can be considered a critical sized bone defect. Defects of 6 mm, 8 mm and 10 mm width are critical up to 12 weeks [49].

Due to the lack of standardized quantitative measurements, a meta-analysis could not be elaborated. Furthermore, there is a lack of homogeneity in reporting histomorphometry data. There is no standardized quantitative measurement applicable for all cases. However, due to the high variability of the included studies, the composition and the use of the biomaterials, their production methods as well as follow-up duration, no decisive statement can be issued regarding the clinical effectiveness of eggshell as a bone regeneration material. There is still place for further research, to design an in vivo animal model with standardized parameters (adoption of critical sized defects, empty control group, analysis), to allow further comparison with similar studies. There is still a need for further research regarding the optimal evaluation for each defect type for the different animal models.

## 5. Conclusions

Within the limitations of this review, eggshell derived grafting materials demonstrated to be osteoconductive in a variety of small animal bone regeneration models, showing better results compared to an empty defect and similar results with Bio-Oss or DMBM. The results show that these biomaterials are promising candidates as bone space fillers. Eggshell particles could be routinely applied to bone defects, to promote tissue healing. Regarding its use as a scaffold for stem cells or growth factors, there is still place for further study.

## Figures and Tables

**Table 1:** Summary of characteristics and main results of studies in rats (*n* = 14).

	Ref	Lot	Defect	Biomaterials	Control	Other Materials/Treatments	Assessment Time, Method(s) Main Findings
**WISTAR RAT**	[5]	10	Mandible angle 5 mm ⌀ full THK bilateral	Eggshell powder	Split mouth empty defect	N/S	8 weeks **Contact Rx**: grafts were dense with a uniform distribution in the bony defect **Histology**: grafts were surrounded by a thin fibrous layer
	[13]	45	Calvaria 6 mm ⌀ full THK bilateral	Eggshell powder Group 1: CMC Group 2: PP + CMC Group 3: ES + PP + CMC	Empty defect	ePTFE membrane for each defect	**6 weeks** **Contact Rx**: centripetal bone regeneration in group I, II; in group III the implant was surrounded by radiolucent border **Histology**: in group 1 and 2 partial bone healing, in group 3 ES displayed marked resorption with fibrous interposition.
	[6]	10	Calvaria 7 mm ⌀ full THK bilateral	Ostrich eggshell implant	Empty defect	N/S	12 weeks **Contact Rx**: the graft was dense but with a surrounding radiolucency. **Histology**: dense capsule lining the outer and inner surface of implant; interposition of fibrous tissue at bone-implant interface.
	[16]	5	Periodontal defect 1.5 × 6 mm bilateral	Eggshell powder Eggshell membrane	Split mouth DMBM Collagen membrane	N/S	6.5 weeks **Histology**: new bone formation at sides of the defect but more bone formation was noted in the control group; more connective tissue was observed in the graft group.
	[17]	18	Calvaria 4 mm ⌀ full THK bilateral	Eggshell brushite cement	Pure brushite cement Empty with collagen	Collagen membrane on the graft	6–12 weeks **microCT**: EB degrades faster than PB; new bone formation from boundaries in the EB group. **Histology**: woven bone was observed for the EB group; PB group surrounded by inflammatory cells. **Immunohistochemistry**: confirmed new bone formation by osteopontin for EB > PB.
	[12]	15	Calvaria 5 mm ⌀ full THK bilateral	Coated eggshell particles (a) CaCO_3_/MgO/CMC2/BMP2 (b) CaCO_3_/MgO/CMC2	(c) Negative control	N/S	8 weeks **microCT**: (a), (b) covered with bone completely. **Fluorescent labeling**: (a) has the best new bone forming capabilities **Histology**: new bone formation in group (a) and (b)
	[15]	40	Calvaria 7 mm ⌀ full THK	(c) Ostrich eggshell powder (d) Ostrich eggshell implant	(a) Empty control group	(b) DMBM	24 weeks **Histology**: group (b) showed the most new bone formation. No difference between groups (**c**) and (d) regarding the new bone formation.
	[14]	45	Calvaria 6 mm ⌀ full THK bilateral	Eggshell powder (c)	Empty (a)	ePTFE membrane for GBR in each group Periosteal graft (b)	2–4–12 weeks **Contact Rx**: group (a) compete closure in 60% of cases; (b) no mineralization; (c) no resorption of the implant. **Histology**: complete closure for 60% of cases (a), no closure for 80% of cases (b) and no closure for all cases in group (c).
SPRAGUE-DAWLEY RAT	[8]	30	Calvaria 5 mm ⌀ full THK bilateral	Eggshell particles (c) Surface modified eggshell particles (d) (ES/CaP-1, ES/CaP-2, ES/CaP-3)	Empty (a)	Bio-Oss (b)	4–8 weeks **Histology:** (a) no defect was filled. (b) bone healing incomplete, graft particles surrounded by fibrous tissue, no resorption of graft. (c) none had complete bone bridging, but two defects showed complete bony closure. (d) completely bone bridging was seen more often than in (b) and (c) **Histomorphometry**: bone formation superior to (d).
	[22]	14	Calvaria 4 mm ⌀ full THK bilateral	Microroughened ostrich eggshell particles (c) CaP coated ostrich eggshell particles (d)	Empty (a)	BioCoral (b)	4 weeks **Histology**: (a) mainly fibrous tissue and bone was formed at defect margins; - new bone formation around grafted particles in the middle area and defect margin for (b), (c), (d).
	[19]	56	Calvaria 8 mm ⌀ full THK	Eggshell nano-hydroxyapatite (b)	Empty (a)	Bio-Oss (c) EHA with CS (d)	6–12 weeks **Histology**: (a) new bone formation at the margins of the defect. (b) increased bone formation compared to (c) and (d). (c) bone formed from the defect margin with few bony islands in the center (d) new bone formation similar to Bio-Oss.
	[20]	30	Calvaria 8 mm ⌀ full THK	Eggshell hydroxyapatite (b)	Empty (a)	sHA (c)	4–8 weeks **Histology**: new bone around de graft particles. (b) (c)—many foreign body multinucleated giant cells were observed surrounding the graft particles. **MicroCT**: bone volume was significantly higher in (b)
	[18]	16	Calvaria 5 mm ⌀ full THK bilateral	Deproteinized ES (a) Hydrothermally treated ES (b)	Empty	Bio-Oss (c)	4–8 weeks **Histology**: (c) new bone was shown in direct apposition to graft particles (b) almost complete bone healing at dura mater side. **Histomorphometry**: (b) showed significantly greater new bone formation than (c)
	[21]	30	4 mm ⌀ half THK 4 defects: -two mandible-two maxilla	ES + carrageenan gel (a) ES + xanthan gel (b) ES powder (c)	Empty	N/S	2–4–6.5 weeks **Histology**: No inflammation at the end of study period. Complete defect healing occurred for group (a).

⌀ = diameter; Contact Rx = contact radiograph; Rx = radiograph, REF. = reference, THK = thickness, N/S = not specified, ePTFE = expanded polytetrafluoroethylene, DMBM = demineralized freeze-dried bone matrix, GBR = guided bone regeneration, microCT = microcomputer tomography, EB = eggshell brushite, BMP 2 = bone morphogenetic protein 2, MgO = magnesium oxide, CaCO_3_ = calcium carbonate, CMC2 = Carboxymethyl chitosan, CT = computer tomography, ESP = eggshell particles, CaP = calcium phosphate, EHA = eggshell hydroxyapatite, CS = calcium sulfate, eHA = eggshell hydroxyapatite, sHA = synthetic hydroxyapatite, ES = eggshell, CMC = carboxymethyl cellulose, PP = pentosan polysulphate, EB = Eggshell brushite cement, PB = Pure brushite cement, ES = eggshell, CaP = Calcium Phosphate, (a), (b), (c), (d) = study groups in the selected study.

**Table 2:** Summary of characteristics and main results of studies in rabbits (*n* = 7).

Ref.	Lot	Defect	Biomaterials(s)	Control	Other Materials/Treatments	Analysis	Assessment Method(s) AND Main Findings
[23]	18	Calvaria 6 mm ⌀ ½ THK (total of 3 defects)	Ostrich eggshell powder	Empty defect (*n* = 1 per rabbit)	Outer shell membrane Inner shell membrane	13	**Contact Rx**: membrane group displayed partial bone healing. In the grafted membrane advanced bone regeneration was present. **Histology**: bone regeneration was seen in the margins of the defect. No statistical difference in bone regeneration between grafted group with membrane or eggshell powder.
[6]	5	Calvaria 15 mm ⌀ full THK	Eggshell implant (interposition graft)	No control	No fixation or osteosynthesis	24	**Contact Rx**: grafts were delineated from the surrounding bone by radiolucency. **Histology**: Similar to Rx, bone condensation was higher at bone-implant interface. There were no signs of remodeling.
[24]	18	Calvaria 6 mm ⌀ ½ THK (*n* = 6 defects in total per rabbit)	Ostrich eggshell particles of different size (grade 1, 2, 3, 4) – each rabbit was grafted with a material per defect (*n* = 5)	Empty defect (*n* = 1 per rabbit)	DBM	4 12 24	**Contact Rx**: bone regeneration at the periphery of the empty defect; small eggshell particles were resorbed faster than larger ones. Higher grade and DBM groups show advanced bone regeneration at 24 weeks. **Histology**: No inflammatory reaction. DBM was completely resorbed and lamellar bone occupied the defect. Smaller eggshell particles were completely resorbed with lamellar bone surrounding it. Connective tissue infiltrated into larger particles of eggshell. **Histomorphometry**: DBM had the largest osseous area. Grade 3 particles followed closely. The empty defect had the least osseous area. Resorption rate of DBM was the highest and the eggshell was resorbed in a size-dependent manner.
[25]	16	Calvaria 8 mm ⌀ Full THK (*n* = 2 defects per rabbit)	Eggshell nanohydroxyapatite	Empty defect (*n* = 1 per rabbit)	nHA+ silk fibroin	4 8	**microCT**: there is a statistically significant difference between the control group and the grafted groups. The nHA+ silk fibroin showed more bone formation than the nHA group. **Histomorphometry**: nHA group showed good bone formation with well-organized lamellar bony islands. The space formed by silk degradation was replaced by new bone but the most area was occupied by poorly degraded biomaterial.
[28]	6	Calvaria 5 × 10 × 1 mm Full THK (*n* = 2 defects per rabbit)	Membrell’s Bonehealth Plus (*n* = 3 rabbits with 6 defects)	Empty defect (*n* = 3 rabbits with 6 defects)	N/S	2	**Histology**: deposition of osteoid newly formed bone trabeculae. No inflammatory cell infiltrate was present. The defects were free from any graft traces. **Histomorphometry**: larger area of newly formed bone was found in the experimental group
[26]	16	Calvaria 8 mm ⌀ Full THK (*n* = 2 defects per rabbit)	Eggshell hydroxyapatite (assigned randomly with the sHA)	Empty defect (*n* = 1 per rabbit)	sHA	4 8	**Histomorphometry**: no difference between the sHA and eHA groups regarding new bone formation. Both had low inflammatory response.
[27]	16	Calvaria 8 mm ⌀ Full THK (*n* = 2 defects per rabbit)	Eggshell hydroxyapatite	Empty defect	N/S	4 8	**microCT**: bone mineral content, bone mineral density, tissue mineral content, tissue mineral density were higher for experimental than control **Histology**: statistically significant more bone formation for the experimental group

THK = thickness, N/S = not specified, EP = eggshell powder, EP = eggshell powder, Rx = radiograph, ESM = eggshell membrane, OSM/ISM = outer/inner shell membrane, DBM = demineralized bone matrix, sHA = synthetic hydroxyapatite, nHA = nano hydroxyapatite, eHA = eggshell hydroxyapatite, microCT = microcomputer tomograph.

**Table 3:** Eggshell originating from Gallus gallus domesticus (Hen) and Struthio camelus (Ostrich) production method and main proprieties.

Ref.	Biomaterial	Production Method and Sterilization	PROPRIETIES
**Eggshell Originating from Gallus Gallus Domesticus (Hen)**			
[5]	Eggshell powder	Eggshell crushed to powder (400–600 µm). Sterilization: Ethylene oxide	-
[13]	Eggshell powder	Eggshell was cleaned, grounded to powder (100–200 µm), and bleached in 6% NaClO 24 h, after which it was washed. Sterilization: Autoclaving	-
[14]	Eggshell powder	Eggshell stripped of membranes, then grounded to powder (400–600 µm). This was bleached in 6% NaClO 24 h. Sterilization: Autoclaving	-
[18]	Deproteinized eggshell (ES-1) Hydrothermally treated eggshell (ES-2, ES-3)	(a) Particulate eggshell was prepared (300 µm), immersed in NaOCl and then HT 300 °C 24 h (ES-1). (b) Hydrothermally treated in phosphate buffered saline at 80 °C (ES-2) (c) Hydrothermally treated in di-ammonium phosphate solution at 150 °C 24 h (ES-2)	**SEM**: ES-2,3 showed microporous surface composed of platelet-like or rod like surface **EDS**: Ca/P atomic ratio Bio-Oss = 1.57 ± 0.41 ES-2 = 1.51 ± 0.20 ES-3 = 1.34 ± 0.09 **XDR**: Calcite peak of CaCO_3_ appeared in ES-1 **FT-IR**: in ES-2 and ES-3 showed sharp splitting of phosphate specific band
[8]	Surface modified natural calcium carbonate eggshell	(a) Fragmented ES were milled (300–500 µm), immersed in 5% NaClO, washed in deionized water and heat treated at 300 °C 24 h (ES). (b) Further treated with phosphate buffer saline (PBS) at 80 °C (ES/CaP-1). Soaked in di-ammonium phosphate solution at 150 °C 24 h (ES/CaP-2) (c) Soaked in a phosphate containing solution at 80 °C (ES/CaP-3). Sterilization: Gamma irradiation	**SEM**: hydrothermally treated eggshell showed different surface morphology—platelet-like, needle-like or rod-like microstructure **EDXA**: atomic ratio of Ca/P showed lower values in the ES/CaP groups than in Bio-Oss **XDR and FT-IR**: indicates partial conversion of the calcite into hydroxyapatite **MTT based assay**: osteoblast cultured with surface modified ES showed significantly higher absorbance compared to Bio-Oss
[19]	Nanohydroxyapatite derived from hen eggshell	Fragmented eggshell (300 µm) was immersed in NaClO. Further they are treated with di-ammonium phosphate solution at 180 °C to make N-HA. prepare a bone substitute composed of outer HA layer and inner CaCO_3_ core. Sterilization: Gamma irradiation	**XDR and FT-IR**: indicate that eggshell (CaCO_3_ in calcite form) converted partially to HA. XDR shows strong peaks of HA and weak peaks of original calcite in N-HA. **FT-IR**: phosphate band resulted from the newly formed HA structure These results indicate that N-HA in composed of outer HA and inner CaCO_3_.
[25]	Nanohydroxyapatite derived from eggshells with or without silk fibroin scaffolds	Raw eggshells underwent calcination at 900 °C 3 h where were crushed and treated with H_3_PO_4_. The powder was milled 10 h in ethanol and pressed at 220 MPa. Then it was sintered at 900 °C 2 h. A Silk fibroin sponge was dipped into a supersaturated solution of N-HA for 1 h.	**SEM**: particles of N-HA showed rectangular shape, some were aggregated; The silk fibroin scaffold was web shaped, with highly porous structures with a round shape at the end. When the N-HA was precipitated into the silk fibroin, the particles were evenly distributed to the surface of the scaffold.
[26]	Hydroxyapatite derived from eggshells	-	**SEM**: sHA had consistent particle size of < 1 µm whereas eHA had > 1 µm The surface roughness of sHA > eHA. Particle size sHA > eHA **FT-IR**: sHA characteristic vibrational model for PO_4_^3−^ eHA PO_4_^3−^ were still detected as a major component and the absorbed water was largely reduced compared to sHA **XDR**: patterns of the sHA and eHA samples matched well the characteristic hexagonal phase of HA. eHA has impure phases of CaO and Ca(OH)_2_.
[21]	Eggshell derived calcium carbonate	Eggshells were cleaned, were crushed to 1 mm porosity of 75%. They were autoclaved. Eggshell derived calcium carbonate combined with carrageenan gel Eggshell derived calcium carbonate combined with xanthan gum gel Eggshell derived calcium carbonate powder	-
[27]	Eggshell derived hydroxyapatite	Eggshells were cleaned and heat treated at 900 °C. The shells were crushed and milled to synthesize calcium phosphate powders.	**SEM**: grain size of 100–200 nm **TEM**: heat treated HA powder consists of two different nanograins: globular (200 nm) and “nanostructured” (70 nm) **XDR**: the main phase is hydroxyapatite. The minor phase of HA powder after milling is hydrogen phosphate, monetite and calcite.
[20]	Hydroxyapatite from eggshells	Eggshells are washed and calcinated at 900 °C for 3 h. Then they are crushed and milled. They are then reacted with phosphoric acid. These mixtures were milled again and sintered at 900 °C.	**SEM**: similar structure with the seashell HA. Average particle size is 0.8 µm × 0.5 µm. **XDR**: the main phase in seashell and eggshell HA was identified as HA. **FT-IR**: characteristic absorption of HA was observed in both samples **ICP-OES**: sodium and strontium contents were higher for the seashell HA. A higher magnesium content was found in eggshell HA.
[17]	Eggshell derived brushite cement (EB)	Eggshells were cleaned, rinsed, dried at 100 °C overnight and powdered. A mixture of eggshell powder (94% CaCO_3_) 1:2 was heated for 12 h to synthesize eggshell derived β-tricalcium phosphate (ETCP). Powder component of pure brushite (PB) cement was made by mixing pure β-tricalcium phosphate (PTCP) with mono calcium phosphate monohydrate (MCPM) 1:1. Powder component of EB was made by mixing ETCP and MCPM.	**XDR**: results confirm the formation of the brushite phase **FT-IR**: presence of the characteristic peaks of brushite **ICP-AES**: it found trace levels of magnesium and sodium other than Ca and P elements. **SEM**: formation of large thin plate-like brushite crystals in the pure brushite cement. In the EB the crystals were found to be smaller in size with irregular morphology.
[16]	Eggshell powder	Eggshells were washed and rinsed. They were crushed to 1 mm diameter. After they were rinsed at 370 °C. Sterilization: Autoclaving	-
[28]	Membrell’s Bonehealth Plus	It is an eggshell based commercially available over the counter dietary supplement for bone health.	**TEM**: particle size < 50 nm
[12]	CaCO_3_/MgO powder	Eggshell powder and magnesium acetate were dissolved and stirred for 3 h and then calcinated at 600 °C for 3 h.	**XDR**: pure phase of MgO and CaCO_3_. The MgO nanoparticles were conjugated well on the surface of eggshell. **SEM**: 10–100 µm particles and good dispersion proprieties. Porous structure of individual particles of 200 to 400 nm. Successful uniform loading of MgO particles (5 nm). TEM: successful loading of MgO particles on the surface of the eggshell.
	CaCO_3_/MgO/CMC/BMP2 scaffold	Carboxymethyl chitosan (CMC) was dissolved in CaCO_3_/MgO solution. BMP2 was added to the mixture.	**FTIR**: amide bone is persistent in the scaffold, existence of carboxymethyl groups, CaCO_3_ **SEM**: interconnected porous architecture, pore size of 50–80 µm **ELISA**: BMP2 could sustainably release from chemically crosslinked scaffold in 4 weeks. **Compression load test**: compression strength higher than CMC scaffold
**Eggshell Originating from Struthio Camelus (Ostrich)**			
[6]	Block 2 mm thick	The shell was immersed in 10% sodium hypochlorite for 24 h. They were washed and autoclaved.	-
[23]	375	Eggshell was ground, washed, dried, and sterilized using ethylene oxide.	-
[22]	300–500	Particles were obtained using mill and sieve, followed by immersion in sodium hypochlorite solution for 48 h. After it was washed. Treatment: Alkaline etching (microroughened-OES) Immersion in supersaturated calcification solution (CaP coated OES) All particles were sterilized using gamma irradiation.	**SEM**: alkaline etching increased surface area; calcium phosphate coating showed platelet-like morphology of crystals.
[24]	700–1500	Small pieces of eggshells were put into Petri dish with glutaraldehyde for 24 h. After they were washed and ground. They were sterilized using ethylene oxide.	-
[15].	20–150 or Block 7 mm Ø	Inner and outer membranes were removed. The eggshells were broken into small pieces of 7 mm diameter. Powder was then prepared with electrical burr. Sterilization was done with ethylene oxide.	- **SEM**: outer surface of ostrich eggshell resembles compact bone and inner surface resembles trabecular bone.

REF = reference, HT = heat treatment, ES-1 = Deproteinized eggshell, ES-2/ES-3 = Hydrothermally treated eggshell, NaClO = sodium hypochlorite, NaOH = sodium hydroxide, H3PO4 = phosphoric acid, SEM = scanning electron microscopy, TEM = transmission electron microscopy, EDS = energy dispersive spectroscopy, EDXA = energy-dispersive X-ray analysis, XDR = X-ray Diffractometry, FT-IT = Fourier-transform infrared absorbance spectra, ICP-OES = plasma optical emission spectroscopy, ICP-AES = inductively coupled plasma atomic emission spectroscopy, Membrell’s Bonehealth Plus = commercially available eggshell supplement, Ø = diameter, CaCO_3_ = calcium carbonate, MgO = magnesium oxide, CMC = carboxymethyl chitosan, BMP2 = bone morphogenetic proteins 2, PBS = phosphate buffer saline, CaP = calcium phosphate, ES = eggshell, Ca = calcium, P = phosphate, N-HA = nanohydroxyapatite, HA = hydroxyapatite, eHA = eggshell derived hydroxyapatite, sHA = synthetic hydroxyapatite, Ca(OH)_2_ = calcium hydroxide, EB = Eggshell derived brushite cement, ETCP = eggshell derived β-tricalcium phosphate, PB = pure brushite, PTCP = pure β-tricalcium phosphate, MCPM = mono calcium phosphate monohydrate, CMC = Carboxymethyl chitosan, OES = ostrich eggshell.

## Supplementary Materials

The following are available online at https://www.mdpi.com/2079-7737/9/12/476/s1, Table S1. Categories to assess the quality of the included studies; Table S2. Study quality assessment—ARRIVE guidelines; Table S3. Risk of bias assessment of the selected studies according to the SYRCLE tool.

## References

[1] Buser D., Dula K., Belser U.C., Hirt H.P., Berthold H. (1995). Localized ridge augmentation using guided bone regeneration. II. Surgical procedure in the mandible. Int. J. Periodontics Restor. Dent..

[2] Hämmerle C.H.F., Karring T. (1998). Guided bone regeneration at oral implant sites. Periodontol. 2000.

[3] Retzepi M., Donos N. (2010). Guided Bone Regeneration: Biological principle and therapeutic applications. Clin. Oral Implants Res..

[4] Benic G.I., Hämmerle C.H.F. (2014). Horizontal bone augmentation by means of guided bone regeneration. Periodontol. 2000.

[5] Dupoirieux L., Pourquier D., Souyris F.F., Surgery M., Prof H., Surgery E., Prof H. (1995). Powdered eggshell: A pilot study on a new bone substitute for use in maxillofacial surgery. J. Cranio-Maxillofac. Surg..

[6] Dupoirieux L. (1999). Ostrich eggshell as a bone substitute: A preliminary report of its biological behaviour in animals—A possibility in facial reconstructive surgery. Br. J. Oral Maxillofac. Surg..

[7] Opris H., Bran S., Dinu C., Baciut M., Prodan D.A., Mester A., Baciut G. (2020). Clinical applications of avian eggshell-derived hydroxyapatite. Bosn. J. Basic Med. Sci..

[8] Park J.-W., Bae S.-R., Suh J.-Y., Lee D.-H., Kim S.-H., Kim H., Lee C.-S. (2008). Evaluation of bone healing with eggshell-derived bone graft substitutes in rat calvaria: A pilot study. J. Biomed. Mater. Res. Part A.

[9] Moher D., Liberati A., Tetzlaff J., Altman D.G. (2009). Preferred Reporting Items for Systematic Reviews and Meta-Analyses: The PRISMA Statement. PLoS Med..

[10] Kilkenny C., Browne W., Cuthill I.C., Emerson M., Altman D.G. (2010). Animal research: Reporting in vivo experiments: The ARRIVE guidelines. Br. J. Pharmacol..

[11] Hooijmans C.R., Rovers M.M., De Vries R.B.M., Leenaars M., Ritskes-Hoitinga M., Langendam M.W. (2014). SYRCLE’s risk of bias tool for animal studies. BMC Med. Res. Methodol..

[12] Huang Y., Ji Y., Kang Z., Li F., Ge S., Yang D.-P., Ruan J., Fan X. (2020). Integrating eggshell-derived CaCO_3_/MgO nanocomposites and chitosan into a biomimetic scaffold for bone regeneration. Chem. Eng. J..

[13] Dupoirieux L., Pourquier D., Picot M.-C.C., Neves M. (1999). The effect of pentosan polysulphate on bone healing of rat cranial defects. J. Craniomaxillofac. Surg..

[14] Dupoirieux L., Neves M., Pourquier D. (2000). Comparison of pericranium and eggshell as space fillers used in combination with guided bone regeneration: An experimental study. J. Oral Maxillofac. Surg..

[15] Uygur S., Ozmen S., Kandal S., Lortlar N., Omeroglu S., Arac M., Cenetoglu S. (2011). Reconstruction of cranial bone defects using Struthio camelus eggshell. J. Craniofac. Surg..

[16] Kavarthapu A., Malaiappan S. (2019). Comparative evaluation of demineralized bone matrix and type II collagen membrane versus eggshell powder as a graft material and membrane in rat model. Indian J. Dent. Res..

[17] Jayasree R., Kumar T.S.S., Venkateswari R., Nankar R.P., Doble M. (2019). Eggshell derived brushite bone cement with minimal inflammatory response and higher osteoconductive potential. J. Mater. Sci. Mater. Med..

[18] Bae S.R., Park J.W., Ahn C.H., Suh J.Y. (2007). In Vivo Evaluation of Hydrothermally Converted Hydroxyapatite as a Bone Graft Substitute. Key Eng. Mater..

[19] Park J.-W., Jang J., Bae S.-R., An C.-H., Suh J.-Y. (2009). Bone formation with various bone graft substitutes in critical-sized rat calvarial defect. Clin. Oral Implants Res..

[20] Lee S.-W., Balázsi C., Balázsi K., Seo D., Kim H.S., Kim C.-H., Kim S.-G. (2014). Comparative Study of hydroxyapatite prepared from seashells and eggshells as a bone graft material. Tissue Eng. Regen. Med..

[21] Uraz A., Gultekin S.E.S.E., Senguven B., Karaduman B., Sofuoglu I.P.I.P., Pehlivan S., Capan Y., Eren K. (2013). Histologic and Histomorphometric Assessment of Eggshell-Derived Bone Graft Substitutes on Bone Healing in Rats. J. Clin. Exp. Dent..

[22] Park J., Lee C., Choi B., Suh J. (2006). Evaluation of natural calcium carbonate with surface modification as a bone graft substitute. Key Eng. Mater..

[23] Durmuş E., Celik I., Ozturk A., Ozkan Y., Aydin M.F. (2003). Evaluation of the potential beneficial effects of ostrich eggshell combined with eggshell membranes in healing of cranial defects in rabbits. J. Int. Med. Res..

[24] Durmuş E., Çelik İ., Aydın M.F., Yıldırım G., Sur E. (2008). Evaluation of the biocompatibility and osteoproductive activity of ostrich eggshell powder in experimentally induced calvarial defects in rabbits. J. Biomed. Mater. Res. Part B Appl. Biomater..

[25] Kweon H., Lee K.-G., Chae C.-H., Balázsi C., Min S.-K., Kim J.-Y., Choi J.-Y., Kim S.G. (2011). Development of nano-hydroxyapatite graft with silk fibroin scaffold as a new bone substitute. J. Oral Maxillofac. Surg..

[26] Lee S.-W., Kim S.-G., Balázsi C., Chae W.-S., Lee H.-O. (2012). Comparative study of hydroxyapatite from eggshells and synthetic hydroxyapatite for bone regeneration. Oral Surg. Oral Med. Oral Pathol. Oral Radiol..

[27] Balázsi C., Gergely G., Balázsi K., Chae C.H., Sim H.Y., Choi J.Y., Kim S.G. (2012). Bone Formation with Nano-Hydroxyapatite from Eggshell. Mater. Sci. Forum.

[28] Salama R., Khashaba M., El Rouby D., El D. (2019). Histomorphometric evaluation of a nano-sized eggshell-containing supplement as a natural alloplast: An animal study. Saudi Dent. J..

[29] Chocholata P., Kulda V., Babuska V. (2019). Fabrication of scaffolds for bone-tissue regeneration. Materials.

[30] Zhang L., Yang G., Johnson B.N., Jia X. (2019). Three-dimensional (3D) printed scaffold and material selection for bone repair. Acta Biomater..

[31] Li S., Li H., Lv G., Duan H., Jiang D., Yan Y. (2016). Influences of degradability, bioactivity, and biocompatibility of the calcium sulfate content on a calcium sulfate/poly(amino acid) biocomposite for orthopedic reconstruction. Polym. Compos..

[32] Takayama T., Todo M., Takano A. (2009). The effect of bimodal distribution on the mechanical properties of hydroxyapatite particle filled poly(L-lactide) composites. J. Mech. Behav. Biomed. Mater..

[33] Zong C., Qian X., Tang Z., Hu Q., Chen J., Gao C., Tang R., Tong X., Wang J. (2014). Biocompatibility and Bone-Repairing Effects: Comparison Between Porous Poly-Lactic-Co-Glycolic Acid and Nano-Hydroxyapatite/Poly(lactic acid) Scaffolds. J. Biomed. Nanotechnol..

[34] Zhang H., Mao X., Du Z., Jiang W., Han X., Zhao D., Han D., Li Q. (2016). Three dimensional printed macroporous polylactic acid/hydroxyapatite composite scaffolds for promoting bone formation in a critical-size rat calvarial defect model. Sci. Technol. Adv. Mater..

[35] Ettl T., Gosau M., Sader R., Reichert T.E. (2012). Jaw cysts—Filling or no filling after enucleation? A review. J. Cranio-Maxillofac. Surg..

[36] Tavangar A., Tan B.B., Venkatakrishnan K. (2011). Synthesis of three-dimensional calcium carbonate nanofibrous structure from eggshell using femtosecond laser ablation. J. Nanobiotechnol..

[37] Arslan Y.E.Y.E., Arslan T.S., Derkus B., Emregul E., Emregul K.C.K.C., Sezgin Arslan T., Derkus B., Emregul E., Emregul K.C.K.C., Arslan T.S. (2017). Fabrication of human hair keratin/jellyfish collagen/eggshell-derived hydroxyapatite osteoinductive biocomposite scaffolds for bone tissue engineering: From waste to regenerative medicine products. Colloids Surf. B Biointerfaces.

[38] Yu H., Zeng X., Deng C., Shi C., Ai J., Leng W. (2018). Exogenous VEGF introduced by bioceramic composite materials promotes the restoration of bone defect in rabbits. Biomed. Pharmacother..

[39] Awaad S.M.N.M.S.H.F.E.M., Naga S.M., Sayed M., El-Maghraby H.F., Awaad M. (2018). Investigation the impact of ZTA addition on the properties of nano biogenic hydroxyapatite. J. Mater. Sci. Mater. Med..

[40] Yu H., Chen Y., Mao M., Liu D., Ai J., Leng W. (2018). PEEK-biphasic bioceramic composites promote mandibular defect repair and upregulate BMP-2 expression in rabbits. Mol. Med. Rep..

[41] Maiti S.K., Shivakumar M.U., Mohan D., Kumar N., Singh K.P. (2018). Mesenchymal Stem Cells of Different Origin-Seeded Bioceramic Construct in Regeneration of Bone Defect in Rabbit. Tissue Eng. Regen. Med..

[42] Liu J., Kerns D.G. (2014). Mechanisms of guided bone regeneration: A review. Open Dent. J..

[43] Mardas N., Chadha V., Donos N. (2010). Alveolar ridge preservation with guided bone regeneration and a synthetic bone substitute or a bovine-derived xenograft: A randomized, controlled clinical trial. Clin. Oral Implants Res..

[44] King’ori A.M. (2011). A Review of the uses of poultry eggshells and shell membranes. Int. J. Poult. Sci..

[45] Faggion C.M., Diaz K.T., Aranda L., Gabel F., Listl S., Alarcón M.A. (2017). The risk of bias of animal experiments in implant dentistry: A methodological study. Clin. Oral Implants Res..

[46] Gomes P.S., Fernandes M.H. (2011). Rodent models in bone-related research: The relevance of calvarial defects in the assessment of bone regeneration strategies. Lab. Anim..

[47] Vajgel A., Mardas N., Farias B.C., Petrie A., Cimões R., Donos N. (2014). A systematic review on the critical size defect model. Clin. Oral Implants Res..

[48] Bosch C., Melsen B., Vargervik K. (1998). Importance of the critical-size bone defect in testing bone-regenerating materials. J. Craniofac. Surg..

[49] Delgado-Ruiz R.A., Calvo-Guirado J.L., Romanos G.E. (2015). Critical size defects for bone regeneration experiments in rabbit calvariae: Systematic review and quality evaluation using ARRIVE guidelines. Clin. Oral Implants Res..

