# Supplementary material for: The Influence of Eggshell on Bone Regeneration in Preclinical In Vivo Studies
Source: Biology (Basel). 2020 Dec 18;9(12):476. doi: 10.3390/biology9120476 (PMC7766478; doi:10.3390/biology9120476)
Supplement: Supplementary file 1 [file biology-09-00476-s001.pdf]

Table S1. Categories to assess the quality of the included studies.

Item	Description	Grade
1	Ethical statement (nature of ethical review permissions or institutional guidelines for care and use of animals)	0 = clearly insufficient<br>1 = possibly sufficient<br>2 = clearly sufficient
2	Experimental procedures (precise details of all procedure performed)	0 = clearly insufficient<br>1 = possibly sufficient<br>2 = clearly sufficient
3	Experimental animals (details of animal used including species, developmental stage or mean age, diagnosis)	0 = clearly insufficient<br>1 = possibly sufficient<br>2 = clearly sufficient
4	Randomization	0 = no<br>1 = unclear<br>2 = yes
5	Allocation concealment	0 = no<br>1 = unclear<br>2 = yes
6	Sample size calculation	0 = no<br>1 = unclear<br>2 = yes
7	Completeness of information	0 = no<br>1 = unclear<br>2 = yes
8	Blinding of the evaluator	0 = no<br>1 = unclear<br>2 = yes
9	Financial conflict of interest	0 = clearly inadequate<br>1 = unclear/possibly adequate<br>2 = clearly adequate

Table S2. Study quality assessment – ARRIVE guidelines

References	Ethical statement	Experimental procedures	Experimental animals	Randomization	Allocation concealment	Sample size calculation	Completeness of information	Blinding of the evaluator	Financial conflict of interest
(Laurent Dupoirieux et al. 1995)	0	2	2	0	1	0	2	1	1
(Laurent Dupoirieux et al. 1999)	0	2	2	0	1	0	2	1	1
(Laurent Dupoirieux, Neves, and Pourquier 2000)	0	2	2	0	1	0	2	1	1
(Bae et al. 2007)	0	2	2	0	1	0	2	1	1
(J.-W. Park et al. 2008)	2	2	2	2	1	0	2	1	1
(J.-W. Park et al. 2009)	2	2	2	0	1	0	2	1	2
(Kweon et al. 2011)	2	2	2	2	1	0	2	1	1
(Lee et al. 2012)	2	2	2	2	1	0	2	1	1
(Uraz et al. 2013)	2	2	2	2	1	0	2	1	1
(Balázsi et al. 2012)	2	2	2	2	1	0	2	1	2
(Lee et al. 2014)	2	2	2	0	1	0	2	1	2
(Jayasree et al. 2019)	2	2	2	2	1	0	2	1	1
(Kavarthapu and Malaiappan 2019)	2	2	2	0	1	2	2	1	2
(Salama et al. 2019)	2	2	2	2	1	0	2	1	2
(Huang et al. 2020)	2	2	2	2	1	0	2	1	2
(L. Dupoirieux 1999)	0	2	2	0	1	0	2	1	1
(E. Durmuş et al. 2003)	2	2	2	0	1	0	2	1	1
(J. Park et al. 2006)	0	2	2	0	1	0	2	1	1
(Ercan Durmuş et al. 2008)	2	2	2	0	1	0	2	1	1
(Uygur et al. 2011)	2	2	2	0	1	0	2	1	1

Table S3. Risk of bias assessment of the selected studies according to the SYRCLE tool

References	Allocation Sequence Generation	Baseline Characteristics	Allocation Concealment	Random Housing	Blinding of Care Giver/ Investigator	Random Outcome Assessment	Blinding of Outcome Assessor	Incomplete Outcome Data Addressed	Free from Selective Outcome	Free from Other Sources of Bias
(Laurent Dupoirieux et al. 1995)	Unclear	Unclear	Unclear	Unclear	No	Unclear	No	Yes	Yes	Yes
(Laurent Dupoirieux et al. 1999)	Unclear	Yes	Unclear	Unclear	No	Unclear	No	Yes	Yes	Yes
(Laurent Dupoirieux, Neves, and Pourquier 2000)	Unclear	Yes	Unclear	Unclear	No	Unclear	No	Yes	Yes	Yes
(Bae et al. 2007)	Unclear	Yes	Unclear	Unclear	No	Unclear	No	Yes	Yes	Yes
(J.-W. Park et al. 2008)	Yes	Yes	Unclear	Unclear	No	Unclear	No	Yes	Yes	Yes
(J.-W. Park et al. 2009)	Unclear	Yes	Unclear	Unclear	No	Unclear	No	Yes	Yes	Yes
(Kweon et al. 2011)	Yes	Yes	Unclear	Unclear	No	Unclear	No	Yes	Yes	Yes
(Lee et al. 2012)	Yes	Yes	Unclear	Unclear	No	Unclear	No	Yes	Yes	Yes
(Uraz et al. 2013)	Yes	Yes	Unclear	Yes	No	Unclear	No	Yes	Yes	Yes
(Balázsi et al. 2012)	Yes	Yes	Unclear	Unclear	No	Unclear	No	Yes	Yes	Yes
(Lee et al. 2014)	Unclear	Unclear	Unclear	Yes	No	Unclear	No	Yes	Yes	Yes
(Jayasree et al. 2019)	Yes	Yes	Unclear	Yes	No	Unclear	No	Yes	Yes	Yes
(Kavarthapu and Malaiappan 2019)	Unclear	Unclear	Unclear	Unclear	No	Unclear	No	Yes	Yes	Yes
(Salama et al. 2019)	Yes	Yes	Unclear	Yes	No	Yes	No	Yes	Yes	Yes
(Huang et al. 2020)	Yes	Yes	Unclear	Yes	No	Unclear	No	Yes	Yes	Yes
(L. Dupoirieux 1999)	Unclear	Unclear	Unclear	Unclear	No	Unclear	No	Yes	Yes	Yes
(E. Durmuş et al. 2003)	Unclear	Unclear	Unclear	Unclear	No	Unclear	No	Yes	Yes	Yes
(J. Park et al. 2006)	Unclear	Yes	Unclear	Yes	No	Unclear	No	Yes	Yes	Yes
(Ercan Durmuş et al. 2008)	Unclear	Unclear	Unclear	Unclear	No	Unclear	No	Yes	Yes	Yes
(Uygur et al. 2011)	Unclear	Yes	Unclear	Yes	No	Unclear	No	Yes	Yes	Yes
